# The Inflammatory Status of Soluble Microenvironment Influences the Capacity of Melanoma Cells to Control T-Cell Responses

**DOI:** 10.3389/fonc.2022.858425

**Published:** 2022-03-28

**Authors:** Gabriela Muller Reche Bogéa, Amandda Évelin Silva-Carvalho, Luma Dayane de Carvalho Filiú-Braga, Francisco de Assis Rocha Neves, Felipe Saldanha-Araujo

**Affiliations:** ^1^Laboratório de Farmacologia Molecular, Faculdade de Ciências da Saúde, Universidade de Brasília, Brasília, Brazil; ^2^Laboratório de Hematologia e Células-Tronco, Faculdade de Ciências da Saúde, Universidade de Brasília, Brasília, Brazil

**Keywords:** melanoma, T-cells, immune response, secretome, checkpoints

## Abstract

The development of immunotherapeutic approaches for the treatment of melanoma requires a better understanding of immunoescape mechanisms of tumor cells and how they interact with other tumor-resident cell types. Here, we evaluated how the conditioned media of resting (rCM) and immune-activated PBMCs (iCM) influence the ability of a metastatic melanoma cell line (MeWo) to control T-cells function. MeWo cells were expanded in RPMI, rCM, or iCM and the secretome generated after cell expansion was identified as MeSec (RPMI), niSec (non-inflammatory), or iSec (inflammatory secretome), respectively. Then, the immunomodulatory potential of such secretomes was tested in PHA-activated PBMCs. iCM induced higher levels of IFN-γ and IL-10 in treated melanoma cells compared to rCM, as well as higher *IDO* and *PD-L1* expression. The iSec was able to inhibit T-cell activation and proliferation. Interestingly, PBMCs treated with iSec presented a reduced expression of the regulators of Th1 and Th2 responses *T-BET* and *GATA-3*, as well as low expression of *IFN-γ*, and co-stimulatory molecules *TIM-3* and *LAG-3*. Importantly, our findings show that melanoma may benefit from an inflammatory microenvironment to enhance its ability to control the T-cell response. Interestingly, such an immunomodulatory effect involves the inhibition of the checkpoint molecules *LAG-3* and *TIM-3*, which are currently investigated as important therapeutic targets for melanoma treatment. Further studies are needed to better understand how checkpoint molecules are modulated by paracrine and cell contact-dependent interaction between melanoma and immune cells. Such advances are fundamental for the development of new therapeutic approaches focused on melanoma immunotherapy.

## Introduction

Cutaneous malignant melanoma (hereafter, melanoma) is a malignant disease of melanocytes with an aggressive clinical pattern, and represents one of the most lethal skin cancers. Progress in identifying the molecular alterations involved in the pathophysiology of melanoma, especially in the gain of proliferative function of tumor cells and in the progression of this cancer, has allowed the search for more effective targeted therapies that can improve the prognosis of melanoma patients (1).

Importantly, both tumor expansion and metastasis are regulated by the cross-talk between tumor cells and the microenvironment in which the tumor is found. The immune system has a primary role in fighting cancer, being able to identify cancer cells and to eliminate them (tumor immunological surveillance). This process involves crucial steps of T-cell activation, followed by the homing of such cells to the melanoma microenvironment, in addition to tumor antigen identification, and tumor cell death induction (2). Therefore, for cancer progression to occur, tumor cells must evade the immune response. To escape immune recognition, tumor cells, such as those of the melanoma, can activate several mechanisms, including the secretion of IDO, IL-10, and TGF-β, the control of T-cells’ function through PDL-1/PD-1 interaction, the generation of regulatory T-cells (Tregs), and the loss of tumor antigens, among others (3, 4). Progress in understanding the immune escape mechanisms of melanoma has led to the development of drugs primarily targeting the checkpoint’s molecules PD-1 and CTLA4. Additional approaches focused on co-inhibitory molecules, such as TIM-3 and LAG-3 have also been explored (5, 6).

Despite recent advances in severe melanoma treatment, which have been mainly based on immunotherapy, cancer relapses and heterogeneous responses among patients are still observed clinically, underscoring the ability of melanoma to escape the immune attack and progress. Therefore, it is essential to better understand the complex and dynamic relationship between melanoma and tumor-resident cells, and how it contributes to disease progression (7).

In this study, we hypothesized that melanoma cells might benefit from the inflammatory environment to better control T-cells function through paracrine mechanisms. For this, we subjected the metastatic site-derived melanoma cell line (MeWo) to the conditioned medium of resting and activated immune cells and determined transcriptional alterations of genes that encode immunomodulatory molecules in this cell line. Next, we evaluated the expression of activation markers, the proliferative capacity, and the expression of checkpoint molecules in PBMCs maintained in the secretome produced by the MeWo lineage previously treated with the conditioned media of the immune cells.

## Methods

### Cell Line, Sample Collection, and PBMCs Obtention

Human melanoma cells from the MeWo cell line (Banco de Células do Rio de Janeiro - BCRJ) were grown in RPMI (Invitrogen) supplemented with 10% (v/v) fetal bovine serum (FBS, Gibco). Peripheral blood samples were obtained from healthy donors at the School of Health Sciences of the University of Brasilia (UnB). Following peripheral blood obtention, PBMCs were isolated by centrifugation using Histopaque (Sigma-Aldrich, USA). The study protocols were approved by the Institutional Ethics Committee and informed consent was obtained from all donors prior to sample collection.

### Experimental Design

**Figure 1:**
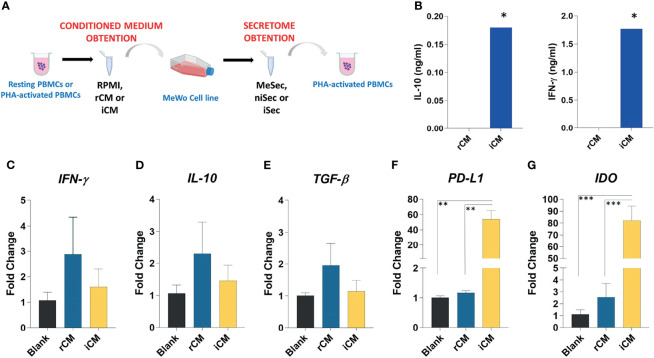
Experimental schedule, characterization of conditioned media and gene expression analysis in MeWo. **(A)** PBMCs were isolated and cultured for 72 hours as resting PBMCs or PHA-activated PBMCs. Further the conditioned medium (RPMI, rCM, and iCM) was obtained and utilized to promote MeWo expansion. When the cell line reached 70% confluence, the media were removed, the cells were washed three times in PBS and cultured for 24 hours in RPMI medium 10% FBS. After this period, the media were aliquoted and used as secretome from melanoma previously submitted to RPMI (MeSec), non-inflammatory (niSec), and inflammatory stimulus (iSec). The immunomodulatory effects of these secretomes were tested in PHA-activated PBMCs. **(B)** IL10 and IFN-γ levels were assessed by ELISA in pooled rCM and iCM. The pools comprised samples obtained from 4 donors. **(C)** *IFN-γ*, **(D)** *IL-10*, **(E)** *TGF-β*, **(F)** *PD-L1* and **(G)** *IDO* expression were assessed in MeWo stimulated by RPMI 10% FBS (Blank), rCM and iCM. Results are presented as mean ± SEM of three independent experiments. *p < 0.05, **p < 0.01, ***p < 0.001.

Experimental design involved three steps: i. PBMC culture and Phytohemagglutinin (PHA) mitogenic stimulation to generate PBMC-conditioned media (named rCM or iCM if produced by resting or immune activated PBMCs, respectively); ii. Treatment of MeWo cells with basal media (Blank), rCM or iCM; iii. Media change and collection of MeWo-conditioned media (named MeSec, niSec or iSec, if produced by MeWo cells kept in basal media, MeWo cells treated with rCM, or MeWo cells treated with iCM, respectively); Finally, RPMI 10% FBS, MeSec, niSec and iSec were used to treat new PHA-activated PBMC samples, in order to assess the paracrine immunomodulatory potential of MeWo (**Figure 1A**).

To do so, first we isolated 1X10^6^ PBMCs from healthy donors (n=4) and cultured them for 72 hours in RPMI 10% FBS or RPMI 10% FBS and 10 µg/ml PHA. The conditioned media obtained from unstimulated PBMCs was named rCM and the conditioned media produced by PHA-stimulated PBMCs was named iCM.

rCM and iCM were then used to treat MeWo cells. To do so, MeWo cells were expanded until reaching 70% confluency. Then, media was changed to either RPMI 10% FBS, rCM or iCM. After 24 hours, cells were washed three times with PBS and fresh media was added. The MeWo-conditioned media was then collected after 18 hours and named MeSec if produced by MeWo cells maintained in basal media only, niSec if produced by MeWo cells treated with the non-inflammatory rCM, or iSec if produced by MeWo cells treated with the inflammatory iCM. Finally, MeSec, niSec and MeSec were diluted 1:1 in RPMI 10% FBS and used to treat new PBMC samples, which were cultured in the presence or absence of PHA stimulus.

### T-cell Activation Analysis

The effects of melanoma secretome on T-cell activation were determined according to the expression of CD69 and HLA-II. For this, activated PBMCs were cultured in MeSec, niSec, or iSec for 24 hours. Then, cells were harvested, stained with anti-CD3-APC, anti-CD69-FITC, or anti-HLA-II-FITC antibodies (eBioscience), and analyzed by flow cytometry, using FlowJo software 10.0.7. Ten thousand events were recorded for each sample.

### T-Cell Proliferation Analysis

In order to determine the influence of melanoma secretome on T-cell proliferation, PBMCs were stained with 5 μM carboxyfluorescein succinimidyl ester (CFSE, Sigma-Aldrich) and activated with 5 µg/ml PHA. Following CFSE staining, activated PBMCs were cultured in MeSec, niSec or iSec for 120 hours. Then, the cells were recovered, incubated with anti-CD3-APC antibody (eBioscience), and used to determine the percentage of CD3^+^/CFSE^+^ cells in each sample by flow cytometry (FacsCalibur, BD Bioscience). Ten thousand events were recorded for each sample, and data were analyzed using FlowJo software 10.0.7.

### Cell Viability Assay

The effect of melanoma secretome on PBMCs viability was investigated by annexin V/PI staining, using flow cytometry. For this, activated PBMCs were cultured in MeSec, niSec, or iSec for 72 hours. After this period, cells were recovered, stained with anti-CD3-APC, annexin V-FITC and Propidium Iodide (PI), according to manufacturer’s instructions. The analyses were performed using the FlowJo software 10.0.7 (FlowJo LLC, USA). Ten thousand events were recorded for each sample. The annexin V^-^/PI^-^ cell population was considered as viable, while annexin V^+^/PI^-^ and annexin V^+^/PI^+^ cell populations were considered as apoptotic.

### Total RNA Isolation and Real Time PCR

Total RNA was extracted from MeWo cells cultured in RPMI 10% FBS (Blank), rCM, or iCM, as well as form PHA-activated PBMCs cultured in MeSec, niSec, or iSec, using Trizol LS (ThermoFisher). After extraction, RNA amount and quality were determined using the NanoDrop 1000 spectrophotometer (NanoDrop). mRNA expression levels of selected genes (**Supplementary Table 1**) were determined by Real-time PCR (Applied Biosystems StepOnePlus™) with SYBR Green Master Mix (Thermo Fisher, USA) combined with primers specific to each gene. The reactions were performed in duplicates, and the relative fold value was obtained by the 2 ^–ΔΔCt^ method.

### Enzyme-Linked Immunosorbent Assay

In order to characterize rCM and iCM, we quantified the levels of IFN-γ and IL-10 by ELISA, following the manufacturer’s instructions (ImmunoTools). The absorbance of each well was measured at 450 nm using the automatic microplate reader DTX 800 Multimode Detector (Beckman Coulter). All samples were analyzed in duplicates.

### Statistical Analysis

Data were reported as mean ± SEM and all analyses were performed using Prism 9 software (GraphPad Software Inc., San Diego, CA, USA). To analyze gaussian distribution of data, the Shapiro-Wilk normality test was used. Statistical significance was calculated using ANOVA (with Tukey’s multiple comparisons test) for variables that followed a normal distribution and Friedman test (with Dunn’s multiple comparisons test) for variables that were not normally distributed. The Mann–Whitney test was used for numerical comparisons between two groups. The value of p < 0.05 was considered statistically significant and significance levels were defined as * p < 0.05; ** p < 0.01; *** p < 0.001; and **** p < 0.0001.

## Results

### PHA Stimulation of PBMCs Induced the Secretion of IFN-γ and IL-10

The conditioned media produced by resting (rCM) or PHA-stimulated PBMCs (iCM) were characterized according to the concentration of IFN-γ and IL-10. As expected, the stimulation of immune cells with PHA induced higher levels of IFN-γ (p=0.01) and IL-10 (p=0.01) in iCM, compared to the rCM (**Figure 1B**).

### iCM Promotes IDO and PD-L1 Overexpression in MeWo Cells

Regardless of the experimental group, no alteration in the expression of *IFN-ɣ*, *IL-10*, and *TGF-β* was detected in MeWo cells (**Figures 1C–E**). However, the exposure of such cells to iCM promoted a significant increase in *PD-L1* expression when compared to Blank group (p=0.002) and rCM (p=0.002). Likewise, we noticed an increase in *IDO* expression in cells cultured in iCM, compared to Blank group (p=0.0004), and rCM (p=0.005) (**Figures 1F, G**). The transcriptional increases of *PD-L1* and *IDO* observed in the MeWo cell line after their culture in iCM indicate a gain in immunomodulatory function in these cells.

### iSec Enhances the Capacity of MeWo to Control T-Cell Activation.

**Figure 2:**
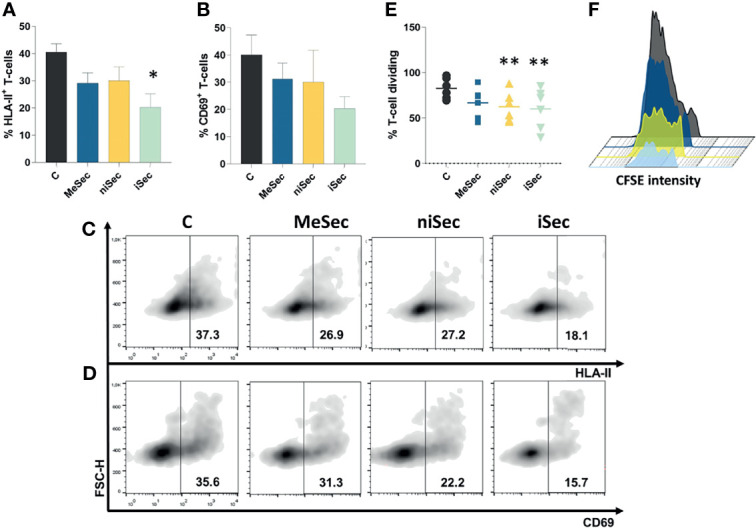
iSec inhibits T-cell activation and proliferation. **(A)** HLA-II expression on T-cells after culture of PHA-activated PBMCs in RPMI **(C)**, MeSec, niSec, and iSec **(B)** CD69 expression on T-cells after culture of PHA-activated PBMCs in RPMI **(C)**, MeSec, niSec, and iSec. **(C, D)** Representative flow cytometric density plots showing expressions of HLA-II and CD69. **(E)** T-cell proliferation after culture of PHA-activated PBMCs in RPMI **(C)**, MeSec, niSec, and iSec. **(F)** Representative CFSE histograms of control group (black), PHA-activated PBMCs cultured in MeSec (dark blue), niSec (yellow), and iSec (light blue). Experiments were carried out with 3-6 PBMCs donors. Results are presented as mean ± SEM. *p < 0.05, **p < 0.01. Ten thousand events were recorded for each sample.

Importantly, iSec promoted a significant reduction of the activation marker HLA-II on T-cell surface (p=0.01). Although the mean expression of CD69 decreased 49.2% when T-cells were exposed to iSec, such an inhibition was not statistically significant (p=0.08) compared to the CD69 levels found on PHA-activated T-cells (**Figures 2A–D**).

### niSec and iSec Enhance the Capacity of MeWo to Control T-Cell Proliferation

In the course of the immune response, following the activation, T-cells proliferate and differentiate into effector cells (8). Interestingly, T-cell proliferation was significantly inhibited after the exposure of these cells to niSec (p=0.005) and iSec (p=0.005) (**Figures 2E, F**), compared to control cells stimulated with PHA in RPMI 10% FBS.

### MeSec, niSec, and iSec Do Not Stimulate T-Cell Apoptosis

**Figure 3:**
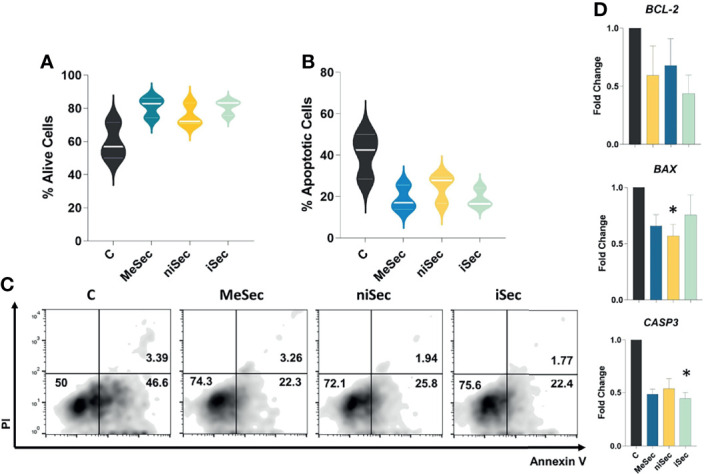
MeWo conditioned media do not stimulate T cell apoptosis. **(A, B)** Percentage of alive (annexin V-/PI-) and apoptotic (annexin V+) T-cells after culture of PHA-activated PBMCs in RPMI **(C)**, MeSec, niSec, and iSec. **(C)** Representative flow cytometric density plots showing expressions of PI and Annexin V in T-cells. **(D)** *BCL-2, BAX* and *CASP3* transcriptional levels in PHA-activated PBMCs cultured in RPMI **(C)**, MeSec, niSec, and iSec. Experiments were carried out with 3 PBMCs donors. Results are presented as mean ± SEM. *p < 0.05. For the flow cytometry analyses, ten thousand events were recorded for each sample.

The exposure of activated T-cells to MeSec, niSec, or iSec did not compromise the viability of these cells (**Figures 3A–C**). Interestingly, at the molecular level, the PBMCs submitted to niSec and iSec showed reduced transcriptional levels of the proapoptotic genes *BAX* (p=0.01) and *CASP3* (p=0.03). No significant changes in transcriptional levels of the anti-apoptotic gene *BCL-2* were detected (**Figure 3D**).

### Transcriptional Effects of MeSec, niSec and iSec in PBMCs

First, we investigated the expression levels of *TIM-3, LAG-3, CTLA-4*, and *PD-1*. Interestingly, TIM-3 expression was significantly reduced in PHA-stimulated PBMCs treated with niSec (p=0.04) and iSec (p=0.01), compared to control cells (stimulated with PHA in RPMI 10% FBS). PHA- stimulated PBMCs exposed to iSec also showed lower expression of *LAG-3* (p=0.01), in comparison with control cells. No statistically significant difference was detected in *CTLA4* and *PD-1* expression. Next, we evaluated the expression of genes encoding the transcription factors *T-BET*, *GATA-3, ROR-γt*, and *FOXP3*. While T-BET levels were significantly reduced in PBMCs cultured in iSec (p=0.007) compared control cells, *GATA-3* presented lower transcriptional levels in PBMCs cultured in niSec (p=0.01) and iSec (p=0.01) compared to control cells. No statistically significant difference was detected regarding *ROR-γt* expression. *FOXP3* expression was found to be reduced in PBMCs cultured in niSec (p=0.04) and iSec (p=0.007) compared to control cells.

**Figure 4:**
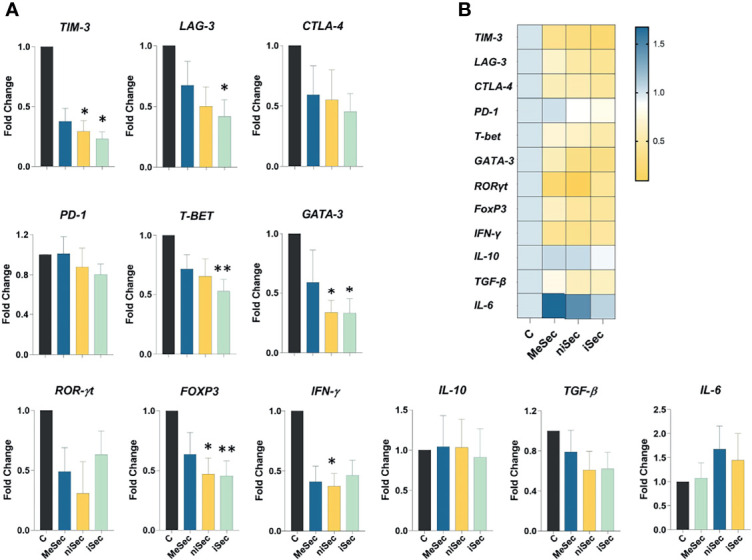
Gene expression analysis in PBMCS. **(A)** *TIM-3, LAG-3, CTLA-4, PD-1, T-BET, GATA-3, ROR-γt, FOXP3, IFN-γ, IL-10*, *TGF-β and IL-6* expression in PHA-activated-PBMCS cultured in RPMI (C, control) MeSec, niSec, and iSec. **(B)** Heat map of these specific genes expressed in PHA-activated-PBMCS cultured in RPMI (C, control), MeSec, niSec, and iSec. Experiments were carried out with 4 PBMCs donors. Results are presented as mean ± SEM. *p < 0.05, **p < 0.01.

Finally, we evaluated the expression levels of *IFN-ɣ, IL-10*, and *TGF-β* in PBMCs cultured in RPMI 10% FBS, MeSec, niSec, and iSec. While no changes in *IL-10*, *TGF-β, and IL-6* were observed, the transcriptional levels of *IFN-ɣ* were significantly reduced in PBMCs exposed to niSec (p=0.01) compared to control cells (**Figure 4**).

## Discussion

Considering the complex interactions that occur in the tumor microenvironment, a better understanding of the relationship between immune and tumor cells creates opportunities for the development of new therapeutic approaches focused on activating the immune system to fight cancer (7). In this work, we demonstrated the impact of the immune response on the capacity of a human metastatic melanoma cell line to exert immunosuppressive effects on T-cells.

Initially, we produced rCM and iCM using resting or PHA-stimulated PBMCs. Our data showed that the use of PHA stimulated the production of IL-10 and mainly IFN-γ by PBMCs. PHA is a widely used T-cell mitogen and stimulates the immune response due to its ability to induce T-cell activation, proliferation and cytokine release including IFN-γ, TNF-α, IL-2, IL-6, IL-10, IL-17, among others (9).

It has been demonstrated that IDO and PD-L1 contribute to cancer-immune escape. IDO expression in metastatic melanoma is associated with an increased number of Treg recruitment to the cancer site and worse patient prognosis (10). Interestingly, our data demonstrate that both IDO and PD-L1 are overexpressed by cancer cells after their exposure to the inflammatory microenvironment of iCM. Indeed, IFN-γ has been reported to induce IDO (10) and PD-L1 (11) expression in melanoma. Besides, it seems that the expression pattern of IFN-γ-related genes correlates with anti-PD-1 therapy response in metastatic melanoma, head and neck carcinoma, and gastric cancer (12). Therefore, a better understanding of how such immunosuppressive molecules are produced and modulated is of particular importance considering that, currently, several drugs based on immunomodulatory mechanisms have been developed for and tested in metastatic melanoma (13).

The immune escape mechanisms attributed to melanoma include the acquisition of a less immunogenic phenotype and the suppression of immune cell function within the tumor microenvironment (7). In agreement with the anti-inflammatory pattern acquired by the MeWo after their exposure to iCM, their secreted products in iSec were able to inhibit HLA-II expression on the T-cell membrane and to reduce CD69 expression in these cells by 49.2%. More importantly, in addition to controlling T-cell activation, iSec also controlled the ability of T-cells to proliferate. Interestingly, a similar ability to control T-cell proliferation has already been reported in the secretome of cervical (14) and head and neck cancer cell lines (15). Thus, our results clearly show that the MeWo cells benefit from an inflammatory milieu, which induces them to create an immunosuppressive microenvironment in a paracrine fashion.

Important advances have been described regarding the capacity of some tumor cells to cause the apoptosis of immune cells through the secretion of soluble factors, including IDO (16) and soluble PD-L1 (17). Considering the effects of niSec and iSec on T-cell proliferation, we investigated whether such an observation involved the induction of PBMC apoptosis. Even though we did not find statistically significant differences, a considerable increase in apoptotic T-cells was observed in the control group, in which PBMCs were activated with PHA. The increase in apoptosis levels in this group was expected as a result of the exacerbated state of T-cell activation (activation-induced cell death-AICD) (16), as evidenced by increased expression of CD69 and HLA-II. Nevertheless, none of the investigated secretomes were able to stimulate T-cell apoptosis, despite the observation of a reduction in the mRNA expression of the proapoptotic genes *BAX* and *CASP3* in PBMCs exposed to niSec and iSec media, respectively.

To better explore the molecular effects of MeWo secretomes on immune cells, we further investigated the expression level of key genes involved in the immune response. We noticed that iCM reduced the mRNA expression of *T-BET* and *GATA-3*, important regulators of Th1 and Th2 response, respectively (18). The stimulation of the Th1 response is important to combat melanoma and is a promising adjuvant strategy in the treatment of this cancer (19). Interestingly, it has been shown that there is an imbalance in Th1/Th2 responses in melanoma and that the activation of the Th2 response is associated with the progression of this cancer (20, 21). However, despite the lower expression of GATA-3 observed in PBMCs treated with iSec, the expression levels of Th2 cytokines *IL-10* and *IL-6* were not reduced.

Consistent with the reduced *T-BET* expression observed by us, the IFN-γ levels were found to be reduced in PBMCs exposed to MeWo secretomes, although such a decrease only reached statistically significant levels in cells treated with niSec. IFN-γ has a crucial role in anti-tumor immunity since this factor can directly mediate tumor rejection and mediate immune cell recruitment to the tumor microenvironment (22). Interestingly, *GATA-3* expression by PHA-stimulated PBMCs was also inhibited by niSec, indicating that the exposure of MeWo cells to the immune cell-conditioned medium, regardless of the state of immunological activation of these cells, would be enough for melanoma to exert an inhibitory effect on the Th2 response.

TGF-β is an important mediator for Tregs since it can regulate the phenotype and function of such cells (23). Several tumor cells produce high levels of TGF-β, regulating Treg generation and maintenance (24). Our data indicate that the cross-talk between MeWo and immune cells does not involve an increased Treg generation as a mechanism of immunomodulation by tumor cells. We did not identify an increase in TGF-β production in MeWo cells after contact with either rCM or iCM, but actually observed reduced transcriptional levels of FOXP3 in PBMCs exposed to niSec and iSec. Although the differences were not statistically significant, they corroborate the observation of reduced *FOXP3* expression, as well as the low transcriptional levels of *TGF-β* detected in PBMCs exposed to niSec and iSec.

Considering that co-inhibitory molecules have been explored to promote anticancer T-cell responses and constitute therapeutic targets in the treatment of melanoma (25), we decided to assess the transcriptional levels of *TIM-3* and *LAG-3* in PBMCS exposed to iSec. Interestingly, the mRNA levels of both genes were found to be significantly reduced in PBMCs exposed to iSec. It is beyond dispute that such an observation still requires further validation at the protein level and also in melanoma samples. Nevertheless, it is possible that - if *TIM-3* and *LAG-3* present a reduced expression in immune cells that were exposed to the secretome of melanoma cells subjected to inflammatory conditions -, they might not be the most effective targets to be explored in the clinic.

Taken together, our observations underscore the relevance of the soluble microenvironment in the cross-talk between melanoma and immune cells. While the milieu produced by resting or activated immune cells induce significant alterations in the MeWo secretome, the melanoma secretome also fosters functional alterations in the immune cells in a context-dependent manner (i.e. whether melanoma cells have been exposed to an inflammatory milieu or not). Therefore, our study reinforces that cell contact-independent mechanisms also influence the complex relationship between cancer and immune system elements. Considering that the evidence accumulated so far regarding the melanoma and immune system interaction is mostly based on experimental settings that involve cell-cell contact, the experimental design proposed herein may contribute for future studies that aim to test new therapeutic approaches in melanoma, to reverse the state of immunosuppression of immune cells. Clinically, our observations allow one to suggest that the paracrine signaling promoted by the melanoma may alter the phenotype and function of tumor-neighboring immune cells that do not interact with cancer cells directly, possibly influencing clinical and treatment outcomes.

## Data Availability Statement

The raw data supporting the conclusions of this article will be made available by the authors, without undue reservation.

## Ethics Statement

The studies involving human participants were reviewed and approved by Comitê de Ética em Pesquisa com Seres Humanos da Faculdade de Ciências da Saúde. The patients/participants provided their written informed consent to participate in this study.

## Author Contributions

GB, AS-C and LF-B designed and carried out the experiments. FN and FS-A analyzed and interpreted data and wrote the manuscript. All authors contributed to the article and approved the submitted version.

## Funding

This study was Funded by DPI/DGP-UnB, Fundação de Amparo à Pesquisa do Distrito Federal (FAPDF) and Conselho Nacional de Desenvolvimento Científico e Tecnológico (CNPq).

## Conflict of Interest

The authors declare that the research was conducted in the absence of any commercial or financial relationships that could be construed as a potential conflict of interest.

## Publisher’s Note

All claims expressed in this article are solely those of the authors and do not necessarily represent those of their affiliated organizations, or those of the publisher, the editors and the reviewers. Any product that may be evaluated in this article, or claim that may be made by its manufacturer, is not guaranteed or endorsed by the publisher.
