# Supplementary material for: The Inflammatory Status of Soluble Microenvironment Influences the Capacity of Melanoma Cells to Control T-Cell Responses
Source: Front Oncol. 2022 Mar 28;12:858425. doi: 10.3389/fonc.2022.858425 (PMC8996246; doi:10.3389/fonc.2022.858425)
Supplement: Supplementary file 1 [file Table_1.docx]

**Table1.** Primer sequences.

| **Gene** | **Primer** | **Sequence (5’- 3’)** |
| --- | --- | --- |
| *GAPDH* | Foward | TCAACGACCACTTTGTCAAGCTCAGCT |
|  | Reverse | GGTGGTCCAGGGGTCTTAC |
| *FOXP3* | Foward | GCACATTCCCAGAGTTCCT |
|  | Reverse | TTGAGTGTCCGCTGCTTC |
| *ROR-γt* | Foward | CTGCTGAGAAGGACAGGGAG |
|  | Reverse | AGTTCTGCTGACGGGTGC |
| *PD-1* | Foward | ATGAAGGCCCCTGGACTAAGA |
|  | Reverse | TGTCCCTGCAGAGAAACACAC |
| *T-BET* | Foward | GGATGCGCCAGGAAGTTTCA |
|  | Reverse | GACTGGAGCACAATCATCTGGG |
| *GATA-3* | Foward | AGCACAGAAGGCAGGGAGTGT |
|  | Reverse | TGATAGAGCCCGCAGGCG |
| *CTLA-4* | Foward | CATCCCTGTCTTCTGCAAAGCAA |
|  | Reverse | CAGTGGCTTTGCCTGGAGAT |
| *LAG-3* | Foward | TCACATTGGCAATCATCACAGTG |
|  | Reverse | CGTTCTTGTCCAGATACTGGAGT |
| *TIM-3* | Foward | CTGCTGCTGCTACTACTTACAAGG |
|  | Reverse | AGACGGGCACGAGGTTCC |
| *BAX* | Foward | CAGACCGTGACCATCTTTGT |
|  | Reverse | GCCTCAGCCCATCTTCTTC |
| *IL-10* | Foward | GGCACCCAGTCTGAGAACAG |
|  | Reverse | ACTCTGCTGAAGGCATCTCG |
| *CASP3* | Foward | CTAGCGGATGGGTGCTATTG |
|  | Reverse | GATACACAGCCACAGGTATGAG |
| *TGF-β* | Foward | GCTGTATTTAAGGACACCGTGC |
|  | Reverse | TGACACAGAGATCCGCAGTC |
| *IDO* | Foward | GGGAAGCTTATGACGCCTGT |
|  | Reverse | CTGGCTTGCAGGAATCAGGA |
| *IFN-γ* | Foward | ACTGTCGCCAGCAGCTAAAA |
|  | Reverse | TATTGCAGGCAGGACAACCA |
| *PD-L1* | Foward | AAACAATTAGACCTGGCTG |
|  | Reverse | TCTTACCACTCAGGACTTG |
